# Automics: an integrated platform for NMR-based metabonomics spectral processing and data analysis

**DOI:** 10.1186/1471-2105-10-83

**Published:** 2009-03-16

**Authors:** Tao Wang, Kang Shao, Qinying Chu, Yanfei Ren, Yiming Mu, Lijia Qu, Jie He, Changwen Jin, Bin Xia

**Affiliations:** 1 Beijing NMR Center, Peking University, Beijing, PR China; 2 College of Life Sciences, Peking University, Beijing, PR China; 3 College of Chemistry and Molecular Engineer, Peking University, Beijing, PR China; 4 Cancer Institute & Hospital, Chinese Academy of Medical Sciences, Beijing, PR China; 5 No. 304 Hospital, Beijing, PR China; 6 Chinese PLA General Hospital, Beijing, PR China

## Abstract

**Background:**

Spectral processing and post-experimental data analysis are the major tasks in NMR-based metabonomics studies. While there are commercial and free licensed software tools available to assist these tasks, researchers usually have to use multiple software packages for their studies because software packages generally focus on specific tasks. It would be beneficial to have a highly integrated platform, in which these tasks can be completed within one package. Moreover, with open source architecture, newly proposed algorithms or methods for spectral processing and data analysis can be implemented much more easily and accessed freely by the public.

**Results:**

In this paper, we report an open source software tool, Automics, which is specifically designed for NMR-based metabonomics studies. Automics is a highly integrated platform that provides functions covering almost all the stages of NMR-based metabonomics studies. Automics provides high throughput automatic modules with most recently proposed algorithms and powerful manual modules for 1D NMR spectral processing. In addition to spectral processing functions, powerful features for data organization, data pre-processing, and data analysis have been implemented. Nine statistical methods can be applied to analyses including: feature selection (Fisher's criterion), data reduction (PCA, LDA, ULDA), unsupervised clustering (K-Mean) and supervised regression and classification (PLS/PLS-DA, KNN, SIMCA, SVM). Moreover, Automics has a user-friendly graphical interface for visualizing NMR spectra and data analysis results. The functional ability of Automics is demonstrated with an analysis of a type 2 diabetes metabolic profile.

**Conclusion:**

Automics facilitates high throughput 1D NMR spectral processing and high dimensional data analysis for NMR-based metabonomics applications. Using Automics, users can complete spectral processing and data analysis within one software package in most cases. Moreover, with its open source architecture, interested researchers can further develop and extend this software based on the existing infrastructure.

## Background

Since Nicholson et al. introduced the terminology [1], metabonomics evolved into a rapid development period. Metabonomics is now widely applied in research areas such as drug toxicology, biomarker discovery, gene function study, functional genomics, natural products research, and molecular pathology etc. [2-5]. Metabonomics studies strongly rely on multiple analytical techniques. These techniques afford a wide range of information for metabolic characterization of biological samples [6-10]. Based on the acquired spectra, data models can be constructed by statistical analysis, pattern recognition methods, and machine learning methods to explain the dynamic activities of metabolites in organisms. Due to the significant quantity and complexity of the spectroscopic data, a major challenge of metabonomics studies is data processing and data interpretation [11]. Therefore, software tools play a significant role in metabonomics studies, and plenty of efforts have been made on software development [12].

Nuclear Magnetic Resonance (NMR) is widely used in metabonomics studies. Compared to other analytical techniques, NMR has the advantage of fully quantitative analysis and minimal requirement for sample preparation [13]. For the "classical" NMR-based metabonomics approach, after NMR experimental data collection, post-experimental data handling including NMR spectral processing, data pre-processing and data analysis, is critical for obtaining good results. To assist these procedures, several software tools have been released, such as: AMIX (Bruker Biospin, Germany), KnowItALL (BIO-RAD, USA), Chenomx NMR Suite (Chenomx, Canada) and Hires [14]. NMRPipe, a widely used traditional NMR data processing software tool [15], also provide some metabonomics related features now. Most of the existing metabonomics tools are commercial products, except Hires, which is free licensed to our knowledge. These software tools provide plenty of functions involving spectral processing, comprehensive identification and quantification of metabolites. Some of these software tools also provide features for basic data analysis, such as principal component analysis (PCA). However, due to the complexity of NMR data and different application purposes, further data analysis procedures, such as filtering out unwanted variations (e.g. background noise, uncorrelated variation in data model, etc.) in a dataset, generating and applying predictive classification or regression models, are usually required. To complete these tasks, researchers usually have to invoke other advanced chemometrics tools or statistical tools. Commercial software packages such as MATLAB (Mathworks, USA), SIMCA-P (Umetrics, Sweden) and SPSS (SPSS, USA) are frequently used by researchers.

In this report, we introduce a new software tool, Automics, the first highly integrated open source software designed specifically for metabonomics to our knowledge. Automics runs on the Microsoft Windows platform, and it is developed with Visual C++. Automics provides features for almost all stages of metabonomics studies, including: NMR spectral processing (high throughput automatic modules and convenient manual modules), data organization, data pre-processing (four data filtering methods), and data analysis (nine data analysis methods), along with other useful functions such as statistical total correlation spectroscopy method (STOCSY), expression calculator and database resource exploring. Automics enables researchers to carry out most of their studies within only one software tool, and thus avoid extensive training on different software tools in order to use them properly. Furthermore, with the keen interest of researchers in developing new algorithms for spectral processing, data pre-processing and data analysis, Automics can serve as a framework for quickly implementing these new data processing algorithms and other useful features, due to its open source architecture. As it provides basic data structures, data management and low-level functions, Automics enables interested developers to focus on kernel algorithmic approaches instead of implementing infrastructure within this framework.

## Implementation

### Overview of software system

**Figure 1:**
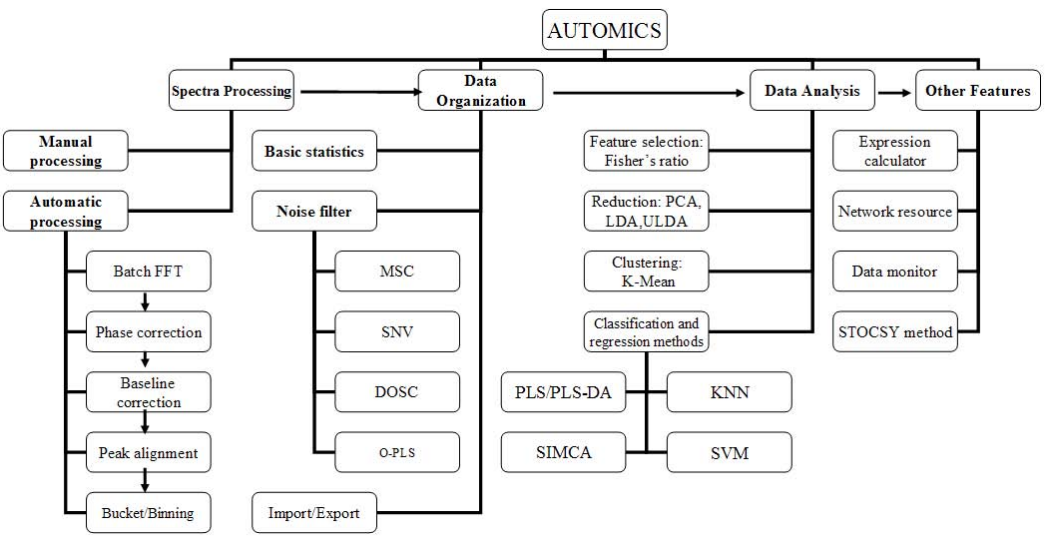
**Functional modules and the workflow of Automics**.

Automics contains modules for almost all stages of post-experimental spectral processing and data handling for NMR-based metabonomics [see Additional file 1]. The features and the workflow of modules are shown in Figure 1. A major goal in the design of Automics is to provide a ready-to-use tool for metabonomics researchers and those who are interested in metabonomics from other fields. Batch spectral processing features, supported by a set of automatic algorithms, have been implemented in order to accelerate the processing procedures and to achieve high efficiency. To facilitate usability, we have developed a user-friendly graphical interface in Automics. Furthermore, to ensure a wide adaptability to different metabonomics applications and to strengthen its data analysis ability, we have integrated nine different data analysis methods into the data analysis module of Automics.

**Figure 2:**
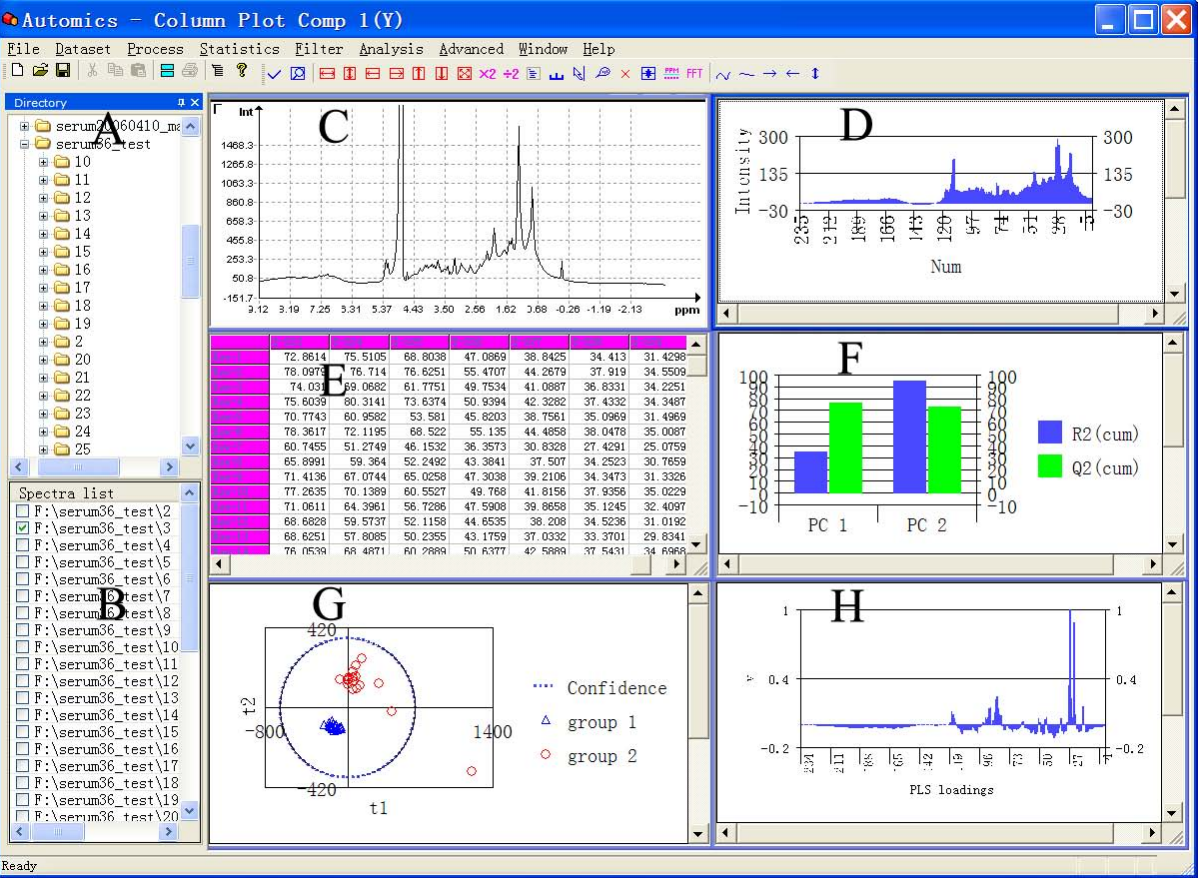
**Screenshot of the main graphical interface**. (A) Directory browser for spectral dataset; (B) List of a selected spectral dataset; (C) Spectral processing window: displaying, moving, zooming and labeling etc. for spectral visualization either in single mode or multiple mode; (D) Column plot of a bucket/binning digitized spectrum; (E) Worksheet of the data organization module; (F) Column plot of the explained variance for R^2 ^(cum) and Q^2 ^(cum); (G) Scatter plot of PLS scores; (H) Column plot of PLS regression coefficients.

The main interface and some of the feature windows of Automics are shown in Figure 2. Automics works in the following steps: NMR spectral processing can be carried out manually in the spectral processing window (Fig. 2A, B, C, D) or automatically using the high throughput custom wizard module. After spectral processing, a data matrix (each row represents a spectrum) is produced by bucket/binning module, and it can be then saved into a text file or exported to a worksheet directly (Fig. 2–E). Based on the worksheet, data pre-processing procedures can be carried out. Finally, data analysis methods are applied to the pre-processed data. The results of analysis and parameters of data models, such as scores and loadings of principal component analysis (PCA) or partial lease squares (PLS) models, can be plotted (Fig. 2F, G, H). In addition, a general plot tool can be launched for visualizing vectors in a data matrix (Fig. 2–D). In order to give the software a good expandability for the future development, Automics was designed with client/server architecture. It is subdivided into several layers. At the very bottom are the foundation classes that implement low-level functions and data structures. The kernel modules that implement data processing algorithms and spectral visualization are built on the low-level dynamic link libraries (DLL).

### Spectra format conversion

Automics takes Bruker (Bruker Biospin, Germany) format raw FIDs and XWIN-NMR processed spectra by default. For NMR data collected on spectrometers from other venders, FID format conversion should be carried out with a conversion module. Before conversion, a meta-data file is first created in a format definition module, which contains parameters for the source format: FID filename, acquisition parameter filename, spectral width (Hz), chemical shift of spectral center (carrier position), observe frequency, optional FID file header size, byte order (little/big endian) and variable type of data points in computer memory (integer, 16/32 bits; float, 32/64 bits). Based on these parameters, Automics can convert raw FIDs of a variety of existing NMR FID formats, such as those from Varian (Varian Inc., USA) and JEOL (JEOL Ltd, Japan), to Bruker FID data format and rewrite them in Bruker style directories.

### Manual spectral processing

Although high throughput automatic spectral processing is one of the major goals and an important feature of Automics, we believe a powerful and convenient manual processing module is still necessary. For situations when the automatic method does not work well (e.g. processing severely distorted spectra), the manual method may be an effective way to correct spectra.

Automics provides an easy to use manual spectral processing module. For spectral visualization, features such as spectral editing, peak labeling, peak information browsing, moving and zooming, are supported. To change the display properties of the visualized spectrum, a dialog can be used to set properties such as line color, line width, line style and background color. For spectral processing, floating tool bars can be used to continuously adjust zero-order and first-order phases for the interactive phase correction, or adjust coefficients of a selected fitting function (polynomial function, sine function and exponential function) for the interactive baseline correction. Other commonly used features, such as referencing, peak picking and spectral derivative, are also supported.

### High throughput automatic spectral processing

**Figure 3:**
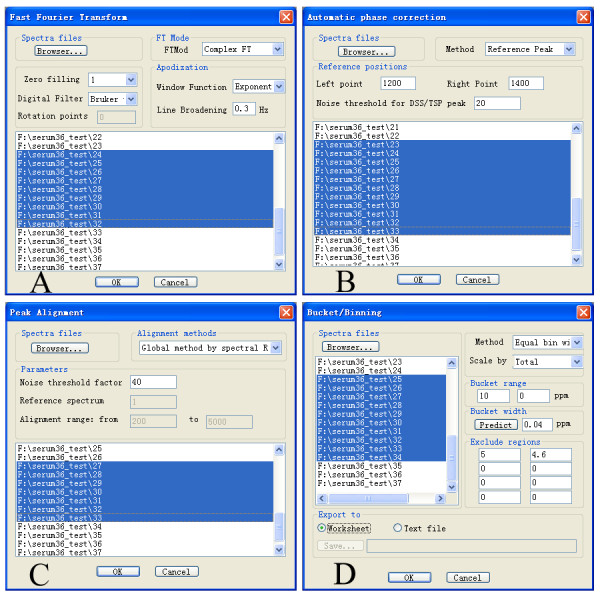
**High throughput automatic spectral processing modules**. (A) Fast Fourier transform; (B) Automatic phase correction; (C) Peak alignment; (D) Bucket/Binning.

1D NMR spectra can be automatically processed in batch mode in five steps: fast Fourier transform (Fig. 3–A), phase correction (Fig. 3–B), baseline correction, peak alignment (Fig. 3–C), and bucket/binning (Fig. 3–D). These features are implemented in five dialogs. They can be launched either individually or through a step-by-step custom wizard from popup menu. The names of selected spectra are shown in a list control, allowing multiple selections.

#### Fast Fourier transform

FFT converts NMR signals from time domain to frequency domain. This module can perform both complex FFT and real FFT. In addition, DC offset, zero filling, window function with a specific line broadening factor and removal of a potential digital filter imposed on FID (such as that from Bruker Avance spectrometer) are also carried out in this module.

#### Automatic phase correction

Traditionally, phase correction is generally accomplished manually by trial and error until the real part of the Fourier transformed spectrum appears globally as a pure NMR absorption spectrum. The corrected spectrum is dependent on one's experience. Several automatic methods have been proposed to estimate zero-order and first-order phases [16-19]. The global methods, such as maximization of the spectrum integral, minimization of the spectrum entropy [20], and a recently patented method by Bruker (it uses a fingerprint of the first derivative as the objective function for the real part of the spectrum and tunes the spectrum until its real part matches its fingerprint the best), require extensively iterative computing and are time consuming. Other methods, such as methods based on dispersion versus absorption relationship (DISPA) [21,22], method based on symmetrizing lines [23] and method based on phase angle measurement from peak areas (PAMPAS) [24], share the common feature that they determine zero-order and first-order phases by linear regression of a set of selected peaks. For these methods, it is critical to find a set of appropriate isolated individual peaks. However, in metabonomics studies, NMR signals from hundreds of metabolites in the samples often cause severe peak overlap, which may affect the accuracy of the phase correction.

Besides two global methods (maximization of the spectrum integral and minimization of the spectrum entropy) implemented in Automics, we have introduced another easier to implement method for automatic phase correction. This method does not require detection of isolated individual peaks and is efficient for processing large quantity of similar spectra in metabonomics studies. Considering a 1D ^1^H NMR spectrum with good baseline, the regions near the two ends of the spectrum are normally free of signals. These regions usually belong to the baseline, and they should have nearly horizontal straight line shape after correction. Based on this principle, enough information can be acquired to calculate the zero-order (*phc0*) and the first-order phases (*phc1*). This method consists of the following steps:

(1) Define two pairs of small regions T_i _and T_j_, T_k _and T_l_(i, j, k and l are the center position of each region in data point) with a certain window length L (for example, 30 data points). T_i _and T_j _belong to the higher frequency baseline region of the spectrum, and T_k_and T_l _are in the lower frequency baseline region of the spectrum. Sum up each region and get their real parts (*R*_*i*_, *R*_*j*_, *R*_*k*_, *R*_*l*_) and imaginary parts (*I*_*i*_, *I*_*j*_, *I*_*k*_, *I*_*l*_);

(2) Determine two phase errors (*θ*_0_, *θ*_1_) at positions of (i + j)/2 and (k + l)/2. Because these four regions all belong to the baseline, and the distance between two regions of each pair is very small, the two regions of each pair should have approximately the same phase errors (with a distance of 100 data points and a 300° first-order phase error, the difference between T_i _and T_j _usually is smaller than 4°). Therefore, T_i _and T_j _should have nearly the same intensity after correction, so do T_k _and T_l_. The two phase errors can be thus calculated with the following equations:

(1) R_i_cos(*θ*_0_)+I_i_sin(*θ*_0_) = R_j_cos(*θ*_0_)+I_j_sin(*θ*_0_)

(2) R_K_cos(*θ*_1_)+I_K_sin(*θ*_1_) = R_1_cos(*θ*_1_)+I_1_sin(*θ*_0_)

Therefore, the phase errors can be expressed as:

(3) *θ*_0 _= arctan((*R*_*i *_- *R*_*j*_)/(*I*_*j *_- *I*_*i*_))+*mπ*

(4) *θ*_1 _= arctan((*R*_*k *_- *R*_*l*_)/(*I*_*l *_- *I*_*k*_))+*nπ*

(3) Calculate the zero-order and first-order phases using the follow equations:

(5) $$\theta_{0}=phc_{0}+\frac{i+j}{2N}phc_{1}$$

(6) $$\theta_{1}=phc_{0}+\frac{k+l}{2N}phc_{1}$$

(4) Correct the spectrum with the determined *phc0 *and *phc1*.

#### Automatic baseline correction

Current version of Automics provides two methods for automatic baseline correction: linear fitting and non-parametric recognition. Linear fitting method uses pre-defined positions of the spectrum to calculate coefficients of a linear function, which are then used to construct a baseline. Our experience has shown that this method works well in most cases, despite its simplicity.

A non-parametric method was implemented with a variant of Sergey's algorithm [25]. It includes two steps. First is baseline recognition. To decide whether a data point belongs to the baseline, the first derivative of the spectrum is calculated, which can be used to distinguish sharp peaks from hump regions in the spectrum and helps to recognize baseline regions. A data point is considered to be on the baseline if the absolute intensity of the corresponding point in the derivative spectrum is below a pre-defined noise threshold. The second step is to construct a smoothed baseline from those recognized data points using a moving convolution window. Then the baseline is subtracted from original spectrum, resulting in baseline corrected spectrum.

### Peak alignment

Frequency shifts due to unstable experimental and instrumental conditions are one of the main sources of unwanted variations for further data analysis. These variations obscure the process of pattern discovery and impede the performance of data analysis. Peak alignment is an essential step to remove effects of such variations from the spectral datasets. Spectral referencing, which sets the inner reference peak (DSS/TSP) of each spectrum to 0 ppm, can be regarded as a simple global method for peak alignment. This method shifts the entire spectrum based on the same reference peak position. Thus, all the spectra with global peak misalignments are well aligned. However, it is not sufficient for correcting individual peak misalignments in spectra, such as those from urine samples with variant solution conditions. Several methods have been proposed to solve this problem. A genetic algorithm can align peaks in automatically selected segments of each spectrum to the corresponding peaks in a pre-selected reference spectrum [26]. A principal component analysis method can identify and adjust individual peak variations through examining the correlation between peak-derivative shapes and the second or higher order principal components (PCs) [27]. As these two methods deal with every data points in the interested regions, both of them are time consuming. Automics implements a fuzzy wrapping method [28]. This method detects the maximal position of peaks in each spectrum and aligns them to a reference spectrum using their similarity determined using a fuzzy Gaussian function. It is more efficient than the above mentioned two approaches due to the reduced data size of processed peaks vector.

### Bucket/binning and normalization

Bucket/binning is a commonly used technique for digitizing a spectrum into a row vector. It has the advantages of minimizing misalignment effects and reducing data dimensionality (usually from several thousand to several hundreds of bins) for further analysis. However, it also leads to a lower data resolution. An extreme case of bucket/binning is that each bin contains a single data point, thus it has a full resolution. However, the quality of data generated in this way highly relies on accurate peak alignment, and this method may bear heavy computing burden such as calculating element-based leave-one-out cross validation.

Automics provides both full resolution (data point) bucket/binning and traditional bucket/binning with equal bin width options (Fig. 3–D). For the second option, we implement a method to determine an appropriate bin width for balancing resolution and dimensionality. First, peak widths for all identified peaks of each spectrum are determined. Then, the average peak width of the "sharper peaks" half is used as bin size. The peak finding and peak width determining are carried out as following:

(1) Noise filtering: use a Savitsky-Golay filtering window to smooth the spectrum by removing high frequency noise with a pre-defined noise threshold.

(2) Peak finding: Among the data points whose intensities are above the threshold, find all maximal points. A maximal point is defined as a point with a number of adjacent consecutive data points on both sides that all have smaller intensities than this point; meanwhile, the intensities of these points on each side are in descending order.

(3) Peak width determining: For each side of a maximal point, the total number of data points whose intensities are in descending order is counted. The sum of the two numbers for both sides is used as peak width for this peak.

In addition to the above mentioned two methods, an intelligent adaptive binning method was also implemented in Automics [29]. This method recursively identifies bin edges in existing bins and requires minimal user input, and it can largely circumvents problems such as the loss of information due to low resolution, the occurrence of artifacts caused by frequency shifts and the presence of noise variables. Generally, normalization of each row vector produced from bucket/binning is required before further data analysis. Four normalization methods are available in Automics: normalizing against the total spectral area, normalizing against the maximum peak area, normalizing against the inner reference peak area, and normalizing against a specific peak area. After bucket/binning and normalization, the produced data matrix can be saved into a comma-delimited text file, or can be exported to a worksheet directly for further analysis in Automics.

### Data organization and data pre-processing

A worksheet module was developed in Automics for data organization. Data pre-processing and data analysis procedures are all based on data in the active worksheet. Automics can import/export data files in text format or Microsoft EXCEL spreadsheet format. Commonly used editing functions and some basic statistical analysis (column based statistics, row based statistics and matrix standardization) are supported.

To remove undesirable systematic variations in the spectroscopic data before data analysis, four commonly used data filter methods were integrated into Automics: multiplicative signal correction (MSC) [30], standard normal variate transform (SNV) [31], direct orthogonal signal correction (DOSC) [32] and orthogonal projections to latent structures (O-PLS) [33]. DOSC is a variant algorithm of the well-known orthogonal signal correction (OSC) [34]. It is a powerful method for removing structured variation which is orthogonal to the response variables (Y matrix), from the observation variables (X matrix). However, in some cases, not all the structured Y-orthogonal variations need to be removed. Only those irrelevant variations that create problems for PLS (or other regression methods) should be removed. O-PLS is a generic hybrid OSC+PLS method which takes the objective of the PLS regression model into account and removes Y-orthogonal variations when necessary.

### Data analysis module

After data pre-processing, data analysis modules can be invoked to analyze the data and build data models. Automics provides nine different pattern recognition methods for data analysis. These methods include feature (variable) selection method (Fisher's criterion (FC) [35]), data reduction method (principal component analysis (PCA), linear discriminant analysis (LDA) [36], uncorrelated linear discriminant analysis (ULDA) [37,38]), unsupervised clustering method (K-Mean Clustering (K-Mean) [39]), and supervised regression and classification methods (partial least squared analysis (PLS) [40,41], K nearest neighbor classification (KNN) [42], soft independent modeling of class analogy (SIMCA) [43] and support vector machine (SVM) [44]).

#### FC

Fisher's criterion method is a feature selection technique. The general purpose of the feature selection is to find significant features (variables) from the original data space in order to produce a better prediction result. Irrelevant features that introduce noises should be eliminated. The importance of an individual feature for discriminating different groups in the training dataset is expressed by the Fisher's ratio, which is the ratio of between-class variance to within-class variance for the training group. A feature with larger Fisher's ratio means that it is more important for classification. Users can select a number of features with the largest Fisher's ratios for further analysis.

#### PCA and PLS

These are two commonly used multivariate analysis techniques in metabonomics studies. The main purpose of PCA is to eliminate the collinear problem and then reduce the dimensionality of the original feature (variable) space. It is an unsupervised method used to reveal the internal structure of datasets in an unbiased way. PLS is a supervised method for regression. The overall goal of PLS is to maximize the covariance between the predictor space and the response space, and then use the predictor matrix to predict responses in the population. With a cutoff for predicted responses, PLS regression can be used for classification and discrimination analysis (PLS-DA). PCA and PLS reveal the variable contribution for the separation between different groups by loadings or regression coefficients.

#### LDA and ULDA

LDA is a well-known technique for the dimension reduction and the feature extraction closely related to PCA. Differing from PCA, LDA is a supervised method. It aims to find an optimal transformation that maps the data into a lower dimensional space with minimized within-class distance and maximized between-class distance, thus achieving the best separation between two or more classes of observations. A variant algorithm, ULDA, was proposed for solving singular problem limitation in LDA. ULDA employs the generalized singular value decomposition method to handle singular data. The advantage of this method is that features in the transformed space are uncorrelated, which makes it attractive for the feature dimension reduction. The work on plasma fatty acid metabolic profiling analysis by Yi et al. [45] showed that a better discrimination is achieved using ULDA feature reduction comparing with that using PCA and PLS data reduction methods, which suggests that ULDA is a good complement for commonly used PCA and PLS methods.

#### K-Mean

K-Mean is an unsupervised method for grouping samples into a fixed number (*k*) of groups in a dataset by their similarity. The similarity is defined by the distance. Several distance measures can be used in K-mean method, such as Euclidean distance, Manhattan distance, or correlation coefficient. K-Mean method implemented in Automics can be used as an initial step to quickly detect outliers and assign class indicators of observations (samples) in metabonomics studies.

#### KNN and SIMCA

KNN and SIMCA are two supervised methods for classification using data similarity. In KNN method, the test dataset is classified by a majority vote of its neighbors, with a sample being assigned to the class most common amongst its *k *nearest neighbors. The *k *value is a positive integer, typically small (such as 3, 5, 7 etc.). If *k *= 1, then the sample is simply assigned to the class of its nearest neighbor (NN). SIMCA works as following: PCA is first performed on each independent group in the dataset, and a sufficient number of principal components are retained to account for most of the variations within each class. Hence, a principal component model is used to represent each class in the dataset. Finally, samples in the test dataset are classified to one of the established models on the basis of their best fit to the respective model.

#### SVM

It was integrated into Automics as another powerful classification tool. SVM is based on rigorous statistical learning theory, and it has been used in a wide range of problems for the classification of datasets such as proteomics data and genomics data. SVM takes a set of features (variables) as input and outputs a classification or a regression vector. It maps input vectors into a higher dimensional feature space using a kernel function. The training procedure leads to the finding of a hyper plane in the feature space, which optimally separates training vectors of two classes. Then, it finds several support vectors that contribute most for the classification. When a new feature vector (sample or row vector in metabonomics) is input, its class membership is predicted on the basis of which side of the plane it maps.

Before invoking data analysis methods, new datasets must be created based on the data in the active worksheet by a dialog interface (Fig. 3–D). This dialog is used to construct training dataset, testing dataset and define variables in them (i.e. variables in X and Y matrix). Four options, central scaling, auto scaling (UV scaling), Pareto scaling and no scaling, are available for users to scale variables (column vectors).

In addition to the implementation of these data analysis algorithms, Automics provides a convenient way to visualize parameters of data models in 2D scatter plot, line plot, or column plot. These parameters usually include scores, loading, explained variances (R^2^, Q^2^), residual matrix, Hotelling's T^2 ^etc. Plot properties (color, legend, title, footnote, scale of an axis etc.) can be conveniently changed. These features in Automics are comparable to those in commercial statistics software.

The performance of many pattern recognition methods are related to the inner data relationship and the data structure. Automics is flexible enough to combine different data pre-processing methods (noise filter, feature selection, dimension reduction) with different classification methods, and produce different classification methods such as FC/KNN, ULDA/KNN, FC/PLS-DA, O-PLS/PLS, and FC/DOSC/SVM etc., to facilitate different metabonomics applications and achieve better results.

## Results and discussion

### Automatic spectral processing

**Figure 4:**
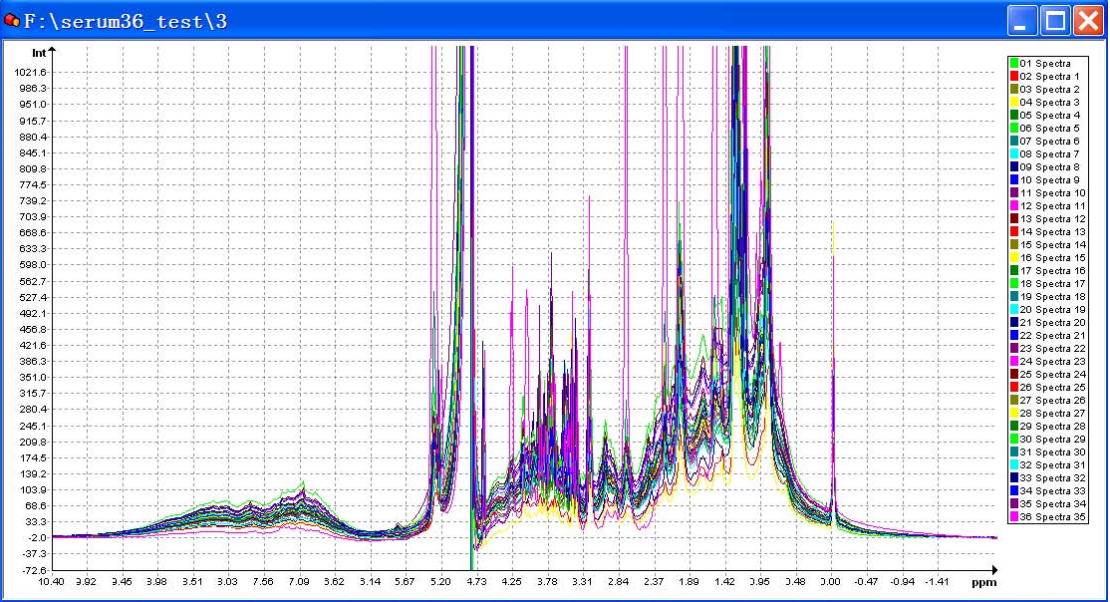
**Overlay display of 1D ^1^H NMR spectra automatically processed by Automics**. The spectra were processed by automatic modules in the following steps: fast Fourier transform, phase correction (new introduced phase correction method), baseline correction (linear fitting) and peak alignment (global shift method).

Automics provides an efficient way for processing a large number of NMR spectra in batch mode by a series of automatic methods, and produces reasonably well corrected spectra. Figure 4 shows an example for displaying multiple processed spectra in Automics. For metabonomics studies, spectral processing results may have a significant impact on data analysis. The automatic spectral processing in Automics has the advantage that all NMR spectra are processed under the same criteria. This will potentially reduce systematic error due to the inconsistency of manual operations.

For several different spectral datasets, the new automatic phase correction algorithm we proposed worked well. We have also examined this method on a set of spectra that contained significant zero-order and first-order phase distortions ranging from 20° to 300°, and we achieved less than 8° errors for the two phase values (data not shown). As our method does not use peaks for determining phases, our method has no weaknesses due to peak shape, digitization rate or peak overlap. The signal-to-noise ratio of a spectrum has little influence on phase determination, owing to our summation procedure. In practice, the baseline regions of a spectrum selected for determining phase errors are not always in a horizontal straight line, sometimes they could be a little tilted and have small slope angles. However, the angles can be determined from a corrected reference spectrum and then be applied to uncorrected spectra as a prior knowledge for compensation. The main drawback of this method is that it relies on a not severely distorted baseline. Nevertheless, the fact that a spectrum can not be phased correctly due to severe baseline problem may indicate an abnormal situation in the NMR experiment, which should not happen very frequently. Therefore, this drawback will not be a significant problem in metabonomics studies.

### A metabonomics application using Automics

We have tested Automics for several application datasets. Here, with an example on the study of metabolic profile in type 2 diabetes, we provide an overview of the validity and the ability of Automics.

#### Sample preparation

Human blood samples were collected from 41 healthy adults and 57 patients with type 2 diabetes mellitus from No. 304 Hospital in Beijing. The ages of patients were between 21 and 79 years (44 ± 17, mean ± STD.). All the samples were collected under the same clinical condition before breakfast. The plasma samples were first allowed to clot in plastic tubes for about 1 hour at room temperature, and then aliquots of serum were collected and stored at -80°C until assayed. Right before the NMR experiment, each serum sample (150 *μ*l) was diluted with 300 *μ*l of 50 mM PBS buffer (pH 7.0), along with addition of 50 *μ*l D_2_O and 3 *μ*l DSS.

#### NMR experiment

All the 1D ^1^H NMR spectra were collected at a temperature of 298 K on a Bruker Avance 600 MHz NMR spectrometer using Bruker pulse sequence NOESY PRESAT, which can be depicted as: RD-90°-t_1_-90°-t_m_-90°-acquisition. RD represents a relaxation delay of 1.5 s during which the water resonance is selectively irradiated, and t_1 _is a fixed time interval. During the mixing time t_m _(150 ms), the water resonance is irradiated for a second time. For each sample, 32 scans were collected into 16 K data points with a spectral width of 9615.4 Hz.

#### Spectral processing

Raw NMR FIDs from 41 healthy samples and 57 diabetic patient samples were processed in Automics using automatic spectral processing module, including Fast Fourier Transform (correction of DC offset, exponential window function with a line broadening factor of 0.3 Hz), phase correction (new method we proposed), baseline correction (linear fitting method) and peak alignment (global shift method). These automatic spectral processing procedures produced a good result (data not shown) and no further manual correction was carried out. All the processed spectra were data reduced to 470 segments between 0.2 ppm and 10.0 ppm using bucket/binning module with a bin width of 0.02 ppm. Due to the strong solvent signal, the spectral region between 4.6 ppm and 5.0 ppm was excluded.

#### PLS analysis together with DOSC and O-PLS

**Figure 5:**
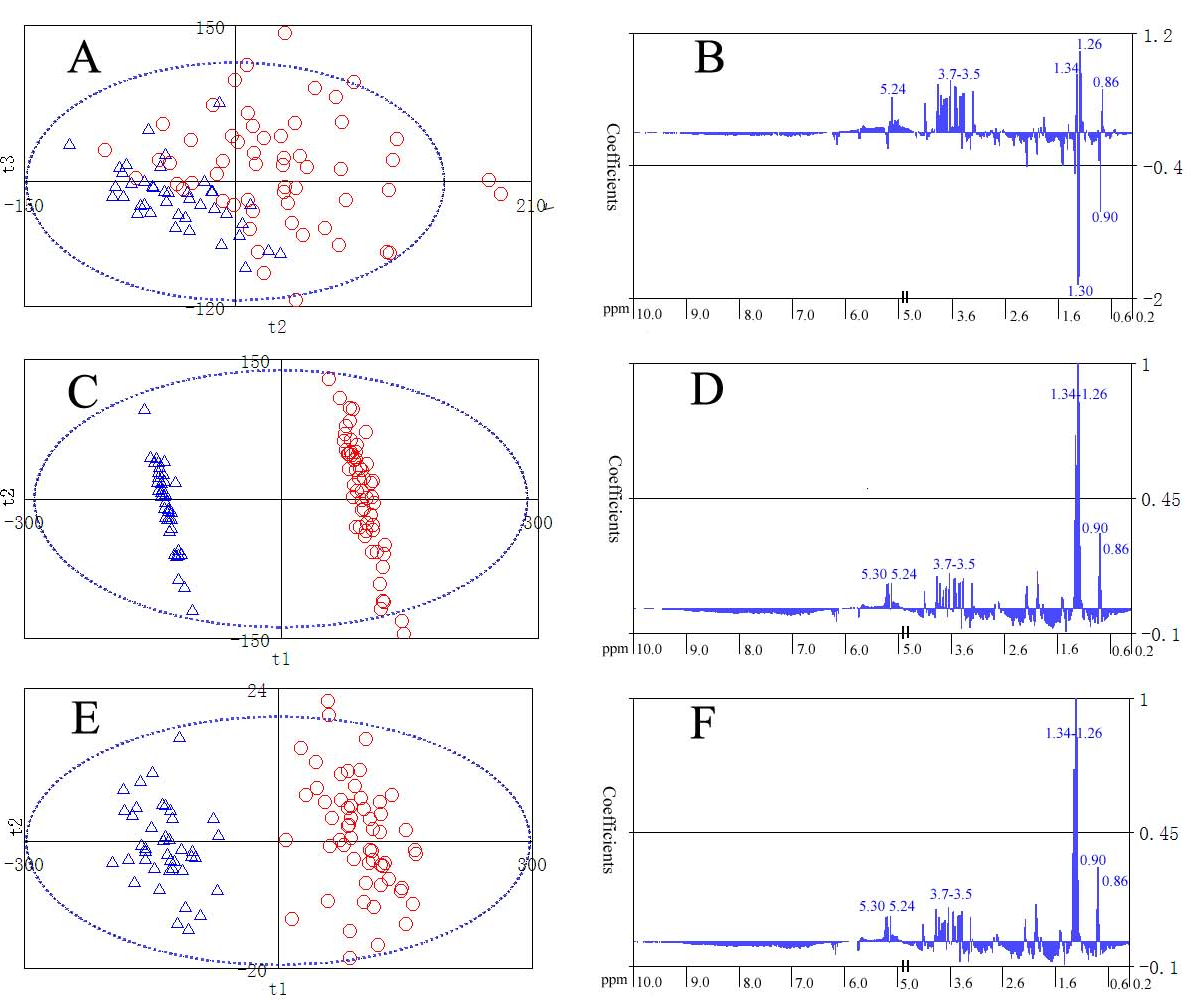
**PLS analysis of type 2 diabetic samples (57) and healthy samples (41) using Automics**. (A) PLS scores show evident clustering between diabetic (○) and healthy (△) samples. The optimal separation occurs in the second and third components (t2, t3). (B) Regression coefficients of the corresponding PLS model. (C) PLS scores after application of DOSC for removal of one orthogonal component. (D) Regression coefficients of the PLS model after application of DOSC. (E) PLS scores after application of O-PLS. Note that the significant improvement for separation is both achieved by DOSC and O-PLS, and now the optimal separation occurs in the first principal component. (F) Regression coefficients of the PLS model after application of O-PLS.

To determine whether it is possible to distinguish healthy and diabetic samples based on the NMR spectra, we carried out PCA and PLS analysis using data analysis module on the mean-centered data. PCA analysis showed that the two groups are severely overlapped. The PLS score plot of the second and the third principal components (t2 and t3) show that some clustering is evident even though there is overlap between the two groups (Fig. 5–A). The regions of the NMR spectrum that most strongly influence the separation between the two groups can be indicated by the regression coefficients. However, the regression coefficients of this model (Fig. 5–B) can not give an exact explanation due to overlap. To improve the performance of the data analysis and filter out unwanted orthogonal variations, DOSC or O-PLS was first applied to the dataset before PLS analysis. After application of DOSC (the first orthogonal component was removed) or O-PLS (orthogonal variations for the first PLS component was removed), the healthy group (in blue color) and the diabetic group (in red color) were well separated by the first PLS component (Fig. 5C, E). Regression coefficients indicate the regions that most strongly influence the separation between the two groups (Fig. 5D, F). Each column of the regression coefficient plots represents a spectral region covering 0.02 ppm. Notice that we have defined a Y class indicator vector as response variables before analysis (1 for diabetic samples and 0 for normal samples), positive regression coefficients indicate that there are relatively higher concentrations of metabolites present in type 2 diabetes, while negative values indicate relatively lower concentration. From regression coefficients (Fig. 5–D, F), it is found that signals significant to the separation mainly lie around 0.86 ppm, 0.90 ppm, 1.26 ppm, 1.30 ppm, 1.34 ppm, a small region near 3.5 ppm, 5.24 ppm and 5.30 ppm. These signals can be tentatively assigned according to previous reports [46,47]. Signals near 0.86 and 0.90 ppm are mainly assigned to CH_3 _groups from fatty acid side chains of lipids, in particular LDL and VLDL; Signals at 1.26 and 1.30 ppm are assigned to (CH_2_)_n _groups from fatty acid side chains of lipids (mainly in VLDL, LDL); the signal at 1.34 ppm is assigned to lactate; the small region near 3.5 ppm should contain a set of signals from CH groups of glucose, sugars, glycerol and amino acids; the signals around 5.24 and 5.30 ppm are from CH groups of *α*-glucose and lipid, respectively. As this was a demo study, we did not investigate other signals. Most of the above mentioned regions have been assigned to glucose, lactate and lipids. They have positive regression coefficients, indicating that concentrations of these metabolites are higher in the diabetic group than those in the healthy group (also by ANOVA, p < 0.01). Diabetes mellitus is a prevalent metabolic disorder disease characterized by elevated blood glucose. It has been demonstrated that the diabetes mellitus is associated with metabolism disorder of lipids and fatty acids [48-50]. Our results are consistent with available knowledge about diabetes mellitus from previous studies and clinical information of the samples.

**Figure 6:**
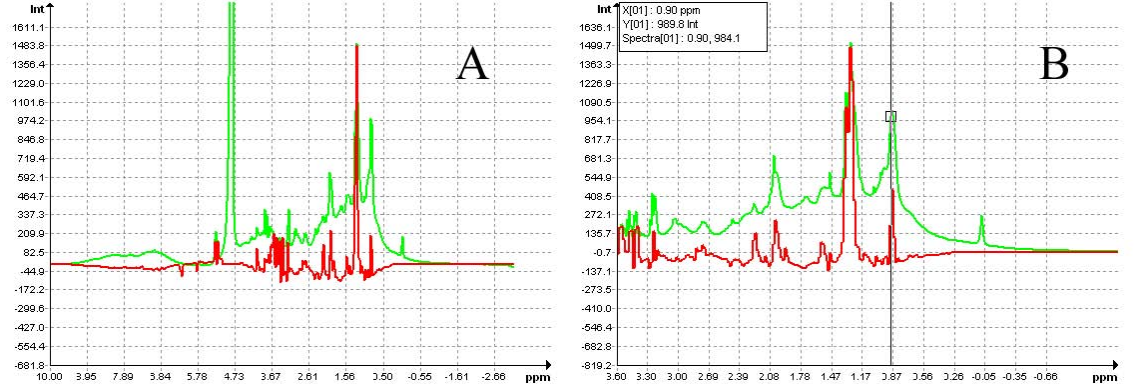
**Feature for overlay display of regression coefficients and the corresponding spectrum in Automics**. (A) Display of the whole spectral region; (B) Display of a zoomed part; (Green curve, NMR spectrum; Red curve, regression coefficients).

In Figure 5, scores and regression coefficients of PLS models are shown in 2D scatter plots and column plots. As researchers usually need to identify corresponding peaks of loadings or regression coefficients, Automics supports overlay display of regression coefficients and the corresponding spectrum in a spectral window (Fig. 6A, B). Researchers can also take advantage of spectral visualization features, such as zooming and peak information exploring, to investigate loadings or regression coefficients conveniently.

### Comparison of different classification methods in Automics

Classification is an important objective in some metabonomics applications. PLS-DA, KNN, SIMCA and SVM implemented in Automics were applied to the dataset for predicting the class membership of unknown samples. Approximately two-thirds of the samples (66/98) were randomly selected to build a training model. The prediction ability of a model was calculated by cross validation. After training, the constructed model was then used to determine the class membership of the remaining one-third samples (testing set, 32/98). We have used different combinations of data pro-processing methods and classification methods to compare their prediction abilities. For example, FC/DOSC/SVM means Fisher's criterion is first used to select an appropriate number of features from the original feature space; then direct orthogonal signal correction is applied to the selected features to filter out unwanted variations; finally, SVM is applied to the corrected data for classification. The results of different combinations are shown in Table 1.

**Table 1:** Comparison of different classification methods

	**Recognition rate**	**Prediction rate**	**Sensitivity**	**Specificity**	**Accuracy rate**
**Ctrl/PLS-DA**	95.5% (63/66)	75.0% (24/32)	91.2% (52/57)	85.4% (35/41)	89.8% (88/98)
**UV/PLS-DA**	98.5% (65/66)	78.1% (25/32)	91.2% (52/57)	92.7% (38/41)	91.8% (90/98)
**DOSC/PLS-DA**	100% (66/66)	84.4% (27/32)	93.0% (53/57)	95.1% (40/41)	94.9% (93/98)
**O-PLS/PLS-DA**	100% (66/66)	81.3% (26/32)	91.2% (52/57)	95.1% (40/41)	93.9% (92/98)
**FC/DOSC/PLS-DA**	98.5% (65/66)	90.6% (29/32)	94.7% (54/57)	97.6% (40/41)	95.9% (94/98)
**KNN (K = 3)**	95.5% (63/66)	71.9% (23/32)	84.2% (48/57)	92.7% (38/41)	87.8% (86/98)
**SIMCA**	90.9% (60/66)	75.0% (24/32)	87.7% (50/57)	82.9% (34/41)	85.7% (84/98)
**FC/KNN (K = 3)**	95.5% (63/66)	81.3% (26/32)	93.0% (53/57)	87.8% (36/41)	90.8% (89/98)
**SVM**	100% (66/66)	81.3% (26/32)	94.7% (54/57)	92.7% (38/41)	93.9% (92/98)
**DOSC/SVM**	100% (66/66)	87.5% (28/32)	94.7% (54/57)	97.6% (40/41)	95.9% (94/98)
**FC/SVM**	100% (66/66)	90.6% (29/32)	96.5% (55/57)	97.6% (40/41)	96.9% (95/98)
**FC/DOSC/SVM**	100% (66/66)	96.9% (31/32)	100% (57/57)	97.6% (40/41)	99.0% (97/98)

Prediction results were from different classification methods (25 healthy and 41 diabetic samples in the training set; 16 healthy and 16 diabetic samples in the testing set). Recognition rate is the correctly classified rate in the training set. Prediction rate is the correctly classified rate in the testing set. Sensitivity is the rate of true positive classified as positive. Specificity is the rate of true negative classified as negative (Ctrl, mean-centered scaling; UV, auto scaling; DOSC, direct orthogonal signal correction; O-PLS, orthogonal projections to latent structures; FC, Fisher's criterion for feature selection).

Without data pre-processing, the number of correctly classified samples in the testing set (prediction rate) decreases in the following order: SVM gives the best result (prediction rate 81.3%); SIMCA and PLS-DA show similar results (prediction rate 75.0%); and KNN produces the worst result (prediction rate 71.9%). With data pre-processing methods (FC was used to select the top 30 significant features from the original variables; DOSC and O-PLS were used to remove orthogonal variations for the first component) applied to the dataset, all the classifiers show better prediction performance. For example, DOSC/PLS-DA gives an improved prediction rate of 84.4%; O-PLS/PLS-DA, DOSC/SVM and FC/SVM also show improved prediction rates of 81.3%, 87.5% and 90.6%, respectively. These demonstrate the general ability of DOSC and O-PLS for removing noise from data set. As shown in Table 1, FC/DOSC/SVM has the best prediction result (prediction rate 96.9%), indicating that combining different data pre-processing techniques can improve the prediction ability. Although O-PLS together with PLS analysis has advantages such as improved interpretability and informative orthogonal variations explain [33], it shows nearly the same prediction rate as DOSC processed PLS model on this dataset.

Whether using data pre-processing or not, SVM gives the best prediction result compared with PLS-DA, KNN and SIMCA on this dataset. The superiority of SVM in prediction suggests that not only well known collinear relationships, but also nonlinear relationships may exist in this dataset. To our knowledge, there are very few applications of SVM in metabonomics studies. The better performance of SVM on our dataset is consistent with the result from Bullinger et al [51]. In their study, SVM also showed a better performance for prediction of breast cancer. Although the classification ability of different classifiers is related to inner data structure of the dataset, we believe SVM is a competitive classifier in metabonomics studies and will be widely used in this field. In this example, SIMCA and KNN did not show better performance than the commonly used PLS-DA classifier, presumably due to that they put a focus on the similarity within a class. In addition, UV scaling on the data has better prediction ability than mean-centered scaling in PLS-DA model (Table 1).

### Other related aspects and the future of Automics

Currently, Automics provides a module for conveniently exploring database resources. This function is helpful when researchers want to explore the structure and chemical shift information of metabolites from available database such as Madison Metabolomics Consortium Database (MMCD) [52] and the Human Metabolome Database (HMDB) [53]. Statistical total correlation spectroscopy (STOCSY) analysis method has also been implemented in Automics. This method takes advantage of the multi co-linearity of the intensity variables in a set of 1D ^1^H spectra to generate a correlation matrix about the intensity correlations among various peaks across the whole dataset [54]. 2D contour plot implemented in Automics can be used to display and analyze the correlation matrix as a pseudo 2D NMR spectrum.

There is still plenty of room for improving the functionality and usability of Automics. For example, some commonly used data analysis approaches such as O2-PLS will be implemented in the near future. A more user-friendly interface is also in our plan for the future development. As Automics was designed with a free open architecture, interested researchers are encouraged to implement new algorithms and extend the software based on the existing infrastructure. We also expect that applications of Automics by metabonomics researchers will help us to get valuable feedbacks and suggestions for improving the platform.

## Conclusion

In this paper, we introduced Automics, the first open source software tool with highly integrated modules specifically designed for NMR-based metabonomics applications. This tool covers almost all stages of the NMR-based metabonomics study workflow. The spectral processing modules in Automics are efficient and convenient for either processing a large number of spectra or processing single spectrum offline without commercial software. In addition, features such as data organization, data pre-processing and a wide range of data analysis techniques for multivariate data analysis, classification and regression have been implemented in Automics. Some of the useful data analysis methods in Automics (such as SVM) are not available in widely used commercial software tools, such as SIMCA-P (Umetrics, Sweden). Automics enables researchers to complete spectral processing and data analysis in one software package. Moreover, Automics could be applied to metabonomics data generated from other analytical techniques (such as mass spectroscopy), owing to its flexible and independent module designs. More details about the usage of Automics can be found in the well-documented HTML help [see Additional file 1].

## Availability and Requirements

**Project name: **Automics

**Project home page: **

**Operating system: **Windows 2000/NT/XP/2003

**Programming language: **Visual C++

**License: **open source under GNU license

## Abbreviations

NMR: Nuclear Magnetic Resonance; MSC: Multiplicative Signal Correction; SNV: Standard Normal Variate Transform; DOSC: Direct Orthogonal Signal Correction; O-PLS: Orthogonal Projection to Latent Structures; FC: Fisher's Criterion; PCA: Principal Component Analysis; LDA: Linear Discriminant Analysis; ULDA: Uncorrelated Linear Discriminant Analysis; PLS: Partial Least Squared; KNN: K Nearest Neighbors; SIMCA: Soft Independent Modeling of Class Analogy; SVM: Support Vector Machine; STOCSY: Statistical Total Correlation Spectroscopy.

## Authors' contributions

BX, CWJ and TW suggested desired features and algorithmic approaches. TW carried out the designing and implementation. KS, QYC, YFR, YMM, LJQ and JH provided samples or experimental data and helped in the evaluation of the package. The online documentation and the manuscript were written by TW with inputs from all co-authors. All authors have read and approved the final manuscript.

## Supplementary Material

Additional File 1 **Software package of Automics.** This compressed file contains executable installation package, source code package, HTML help file, test dataset and readme file of Automics. The latest version is available at Automics homepage . Click here for file
