# Supplementary material for: Automics: an integrated platform for NMR-based metabonomics spectral processing and data analysis
Source: BMC Bioinformatics. 2009 Mar 16;10:83. doi: 10.1186/1471-2105-10-83 (PMC2666662; doi:10.1186/1471-2105-10-83)
Supplement: Additional File 1 — Software package of Automics. This compressed file contains executable installation package, source code package, HTML help file, test dataset and readme file of Automics. The latest version is available at Automics homepage . [file 1471-2105-10-83-S1.zip › Automics_software/Automics_source.zip!/Automics_source/Automics.plg]

```
# Build Log


### --------------------Configuration: Automics - Win32 Release--------------------


### Command Lines

Creating command line "rc.exe /l 0x409 /fo"Release/Automics.res" /d "NDEBUG" /d "_AFXDLL" "F:\±à³Ì¿ª·¢\Mat_C\Automics\Automics.rc"" 
Creating temporary file "C:\DOCUME~1\angell\LOCALS~1\Temp\RSP5B0E.tmp" with contents
[
/nologo /MD /W3 /GX /O2 /I "..\..\GuiLib" /D "WIN32" /D "NDEBUG" /D "_WINDOWS" /D "_AFXDLL" /D "_MBCS" /Fp"Release/Automics.pch" /Yu"stdafx.h" /Fo"Release/" /Fd"Release/" /FD /c 
"F:\±à³Ì¿ª·¢\Mat_C\Automics\AddResource.cpp"
"F:\±à³Ì¿ª·¢\Mat_C\Automics\AddSpectra.cpp"
"F:\±à³Ì¿ª·¢\Mat_C\Automics\AixProperty.cpp"
"F:\±à³Ì¿ª·¢\Mat_C\Automics\AutoAlignment.cpp"
"F:\±à³Ì¿ª·¢\Mat_C\Automics\AutoBaseline.cpp"
"F:\±à³Ì¿ª·¢\Mat_C\Automics\Automics.cpp"
"F:\±à³Ì¿ª·¢\Mat_C\Automics\AutomicsDoc.cpp"
"F:\±à³Ì¿ª·¢\Mat_C\Automics\AutomicsView.cpp"
"F:\±à³Ì¿ª·¢\Mat_C\Automics\AutoPhase.cpp"
"F:\±à³Ì¿ª·¢\Mat_C\Automics\BaselineCorrection.cpp"
"F:\±à³Ì¿ª·¢\Mat_C\Automics\BCMenu.cpp"
"F:\±à³Ì¿ª·¢\Mat_C\Automics\BrowerResource.cpp"
"F:\±à³Ì¿ª·¢\Mat_C\Automics\BucketDlg.cpp"
"F:\±à³Ì¿ª·¢\Mat_C\Automics\ChartProperty.cpp"
"F:\±à³Ì¿ª·¢\Mat_C\Automics\ChildFrm.cpp"
"F:\±à³Ì¿ª·¢\Mat_C\Automics\ColumnStatistics.cpp"
"F:\±à³Ì¿ª·¢\Mat_C\Automics\CommonUtils.cpp"
"F:\±à³Ì¿ª·¢\Mat_C\Automics\DirTreeCtrl.cpp"
"F:\±à³Ì¿ª·¢\Mat_C\Automics\DisplayVariables.cpp"
"F:\±à³Ì¿ª·¢\Mat_C\Automics\Expression.cpp"
"F:\±à³Ì¿ª·¢\Mat_C\Automics\ExpressionCal.cpp"
"F:\±à³Ì¿ª·¢\Mat_C\Automics\FisherCriterion.cpp"
"F:\±à³Ì¿ª·¢\Mat_C\Automics\font.cpp"
"F:\±à³Ì¿ª·¢\Mat_C\Automics\font1.cpp"
"F:\±à³Ì¿ª·¢\Mat_C\Automics\FormatConvert.cpp"
"F:\±à³Ì¿ª·¢\Mat_C\Automics\FormatDefinition.cpp"
"F:\±à³Ì¿ª·¢\Mat_C\Automics\FourierTransform.cpp"
"F:\±à³Ì¿ª·¢\Mat_C\Automics\GuiFolderVisio.cpp"
"F:\±à³Ì¿ª·¢\Mat_C\Automics\HtmlResource.cpp"
"F:\±à³Ì¿ª·¢\Mat_C\Automics\ImportFile.cpp"
"F:\±à³Ì¿ª·¢\Mat_C\Automics\KMean.cpp"
"F:\±à³Ì¿ª·¢\Mat_C\Automics\KNNClass.cpp"
"F:\±à³Ì¿ª·¢\Mat_C\Automics\LabelProperty.cpp"
"F:\±à³Ì¿ª·¢\Mat_C\Automics\LDA.cpp"
"F:\±à³Ì¿ª·¢\Mat_C\Automics\MainFrm.cpp"
"F:\±à³Ì¿ª·¢\Mat_C\Automics\MatrixScale.cpp"
"F:\±à³Ì¿ª·¢\Mat_C\Automics\ModelSummary.cpp"
"F:\±à³Ì¿ª·¢\Mat_C\Automics\MoveSpectra.cpp"
"F:\±à³Ì¿ª·¢\Mat_C\Automics\MSCCorrection.cpp"
"F:\±à³Ì¿ª·¢\Mat_C\Automics\mschart.cpp"
"F:\±à³Ì¿ª·¢\Mat_C\Automics\msflexgrid.cpp"
"F:\±à³Ì¿ª·¢\Mat_C\Automics\MyEdit.cpp"
"F:\±à³Ì¿ª·¢\Mat_C\Automics\MyListCtrl.cpp"
"F:\±à³Ì¿ª·¢\Mat_C\Automics\MySpin.cpp"
"F:\±à³Ì¿ª·¢\Mat_C\Automics\NewModel.cpp"
"F:\±à³Ì¿ª·¢\Mat_C\Automics\OPLS.cpp"
"F:\±à³Ì¿ª·¢\Mat_C\Automics\OSCCorrection.cpp"
"F:\±à³Ì¿ª·¢\Mat_C\Automics\PCA.cpp"
"F:\±à³Ì¿ª·¢\Mat_C\Automics\PeaklistDlg.cpp"
"F:\±à³Ì¿ª·¢\Mat_C\Automics\PhaseCorrection.cpp"
"F:\±à³Ì¿ª·¢\Mat_C\Automics\picture.cpp"
"F:\±à³Ì¿ª·¢\Mat_C\Automics\PlotProperty.cpp"
"F:\±à³Ì¿ª·¢\Mat_C\Automics\PlotSettingsProperty.cpp"
"F:\±à³Ì¿ª·¢\Mat_C\Automics\PrincipleComp.cpp"
"F:\±à³Ì¿ª·¢\Mat_C\Automics\ProgDlg.cpp"
"F:\±à³Ì¿ª·¢\Mat_C\Automics\ProjectStatus.cpp"
"F:\±à³Ì¿ª·¢\Mat_C\Automics\rowcursor.cpp"
"F:\±à³Ì¿ª·¢\Mat_C\Automics\RowStatistics.cpp"
"F:\±à³Ì¿ª·¢\Mat_C\Automics\ScoreProperty.cpp"
"F:\±à³Ì¿ª·¢\Mat_C\Automics\Simca.cpp"
"F:\±à³Ì¿ª·¢\Mat_C\Automics\SortStringArray.cpp"
"F:\±à³Ì¿ª·¢\Mat_C\Automics\SpectraLoading.cpp"
"F:\±à³Ì¿ª·¢\Mat_C\Automics\SpectraParameter.cpp"
"F:\±à³Ì¿ª·¢\Mat_C\Automics\StatisticalTOCSY.cpp"
"F:\±à³Ì¿ª·¢\Mat_C\Automics\svm.cpp"
"F:\±à³Ì¿ª·¢\Mat_C\Automics\SVMTest.cpp"
"F:\±à³Ì¿ª·¢\Mat_C\Automics\SVMTrain.cpp"
"F:\±à³Ì¿ª·¢\Mat_C\Automics\SVNCorrection.cpp"
"F:\±à³Ì¿ª·¢\Mat_C\Automics\ULDA.cpp"
"F:\±à³Ì¿ª·¢\Mat_C\Automics\vcaxis.cpp"
"F:\±à³Ì¿ª·¢\Mat_C\Automics\vcaxisgrid.cpp"
"F:\±à³Ì¿ª·¢\Mat_C\Automics\vcaxisscale.cpp"
"F:\±à³Ì¿ª·¢\Mat_C\Automics\vcaxistitle.cpp"
"F:\±à³Ì¿ª·¢\Mat_C\Automics\vcbackdrop.cpp"
"F:\±à³Ì¿ª·¢\Mat_C\Automics\vcbrush.cpp"
"F:\±à³Ì¿ª·¢\Mat_C\Automics\vccategoryscale.cpp"
"F:\±à³Ì¿ª·¢\Mat_C\Automics\vccolor.cpp"
"F:\±à³Ì¿ª·¢\Mat_C\Automics\vccoor.cpp"
"F:\±à³Ì¿ª·¢\Mat_C\Automics\vcdatagrid.cpp"
"F:\±à³Ì¿ª·¢\Mat_C\Automics\vcdatapoint.cpp"
"F:\±à³Ì¿ª·¢\Mat_C\Automics\vcdatapointlabel.cpp"
"F:\±à³Ì¿ª·¢\Mat_C\Automics\vcdatapoints.cpp"
"F:\±à³Ì¿ª·¢\Mat_C\Automics\vcfill.cpp"
"F:\±à³Ì¿ª·¢\Mat_C\Automics\vcfont.cpp"
"F:\±à³Ì¿ª·¢\Mat_C\Automics\vcfootnote.cpp"
"F:\±à³Ì¿ª·¢\Mat_C\Automics\vcframe.cpp"
"F:\±à³Ì¿ª·¢\Mat_C\Automics\vcintersection.cpp"
"F:\±à³Ì¿ª·¢\Mat_C\Automics\vclabel.cpp"
"F:\±à³Ì¿ª·¢\Mat_C\Automics\vclabels.cpp"
"F:\±à³Ì¿ª·¢\Mat_C\Automics\vclcoor.cpp"
"F:\±à³Ì¿ª·¢\Mat_C\Automics\vclegend.cpp"
"F:\±à³Ì¿ª·¢\Mat_C\Automics\vclight.cpp"
"F:\±à³Ì¿ª·¢\Mat_C\Automics\vclightsource.cpp"
"F:\±à³Ì¿ª·¢\Mat_C\Automics\vclightsources.cpp"
"F:\±à³Ì¿ª·¢\Mat_C\Automics\vclocation.cpp"
"F:\±à³Ì¿ª·¢\Mat_C\Automics\vcmarker.cpp"
"F:\±à³Ì¿ª·¢\Mat_C\Automics\vcpen.cpp"
"F:\±à³Ì¿ª·¢\Mat_C\Automics\vcplot.cpp"
"F:\±à³Ì¿ª·¢\Mat_C\Automics\vcplotbase.cpp"
"F:\±à³Ì¿ª·¢\Mat_C\Automics\vcrect.cpp"
"F:\±à³Ì¿ª·¢\Mat_C\Automics\vcseries.cpp"
"F:\±à³Ì¿ª·¢\Mat_C\Automics\vcseriescollection.cpp"
"F:\±à³Ì¿ª·¢\Mat_C\Automics\vcseriesmarker.cpp"
"F:\±à³Ì¿ª·¢\Mat_C\Automics\vcseriesposition.cpp"
"F:\±à³Ì¿ª·¢\Mat_C\Automics\vcshadow.cpp"
"F:\±à³Ì¿ª·¢\Mat_C\Automics\vcstatline.cpp"
"F:\±à³Ì¿ª·¢\Mat_C\Automics\vctextlayout.cpp"
"F:\±à³Ì¿ª·¢\Mat_C\Automics\vctick.cpp"
"F:\±à³Ì¿ª·¢\Mat_C\Automics\vctitle.cpp"
"F:\±à³Ì¿ª·¢\Mat_C\Automics\vcvaluescale.cpp"
"F:\±à³Ì¿ª·¢\Mat_C\Automics\vcview3d.cpp"
"F:\±à³Ì¿ª·¢\Mat_C\Automics\vcwall.cpp"
"F:\±à³Ì¿ª·¢\Mat_C\Automics\vcweighting.cpp"
"F:\±à³Ì¿ª·¢\Mat_C\Automics\Wizard.cpp"
"F:\±à³Ì¿ª·¢\Mat_C\Automics\Worksheet.cpp"
"F:\±à³Ì¿ª·¢\Mat_C\Automics\XGraph2DView.cpp"
"F:\±à³Ì¿ª·¢\Mat_C\Automics\XGraphTestView.cpp"
]
Creating command line "cl.exe @C:\DOCUME~1\angell\LOCALS~1\Temp\RSP5B0E.tmp" 
Creating temporary file "C:\DOCUME~1\angell\LOCALS~1\Temp\RSP5B0F.tmp" with contents
[
/nologo /MD /W3 /GX /O2 /I "..\..\GuiLib" /D "WIN32" /D "NDEBUG" /D "_WINDOWS" /D "_AFXDLL" /D "_MBCS" /Fp"Release/Automics.pch" /Yc"stdafx.h" /Fo"Release/" /Fd"Release/" /FD /c 
"F:\±à³Ì¿ª·¢\Mat_C\Automics\StdAfx.cpp"
]
Creating command line "cl.exe @C:\DOCUME~1\angell\LOCALS~1\Temp\RSP5B0F.tmp" 
Creating temporary file "C:\DOCUME~1\angell\LOCALS~1\Temp\RSP5B10.tmp" with contents
[
.\bin\htmlhelp.lib /nologo /subsystem:windows /incremental:no /pdb:"bin/Automics.pdb" /machine:I386 /out:".\bin/Automics.exe" /libpath:"..\..\bin" 
".\Release\AddResource.obj"
".\Release\AddSpectra.obj"
".\Release\AixProperty.obj"
".\Release\AutoAlignment.obj"
".\Release\AutoBaseline.obj"
".\Release\Automics.obj"
".\Release\AutomicsDoc.obj"
".\Release\AutomicsView.obj"
".\Release\AutoPhase.obj"
".\Release\BaselineCorrection.obj"
".\Release\BCMenu.obj"
".\Release\BrowerResource.obj"
".\Release\BucketDlg.obj"
".\Release\ChartProperty.obj"
".\Release\ChildFrm.obj"
".\Release\ColumnStatistics.obj"
".\Release\CommonUtils.obj"
".\Release\DirTreeCtrl.obj"
".\Release\DisplayVariables.obj"
".\Release\Expression.obj"
".\Release\ExpressionCal.obj"
".\Release\FisherCriterion.obj"
".\Release\font.obj"
".\Release\font1.obj"
".\Release\FormatConvert.obj"
".\Release\FormatDefinition.obj"
".\Release\FourierTransform.obj"
".\Release\GuiFolderVisio.obj"
".\Release\HtmlResource.obj"
".\Release\ImportFile.obj"
".\Release\KMean.obj"
".\Release\KNNClass.obj"
".\Release\LabelProperty.obj"
".\Release\LDA.obj"
".\Release\MainFrm.obj"
".\Release\MatrixScale.obj"
".\Release\ModelSummary.obj"
".\Release\MoveSpectra.obj"
".\Release\MSCCorrection.obj"
".\Release\mschart.obj"
".\Release\msflexgrid.obj"
".\Release\MyEdit.obj"
".\Release\MyListCtrl.obj"
".\Release\MySpin.obj"
".\Release\NewModel.obj"
".\Release\OPLS.obj"
".\Release\OSCCorrection.obj"
".\Release\PCA.obj"
".\Release\PeaklistDlg.obj"
".\Release\PhaseCorrection.obj"
".\Release\picture.obj"
".\Release\PlotProperty.obj"
".\Release\PlotSettingsProperty.obj"
".\Release\PrincipleComp.obj"
".\Release\ProgDlg.obj"
".\Release\ProjectStatus.obj"
".\Release\rowcursor.obj"
".\Release\RowStatistics.obj"
".\Release\ScoreProperty.obj"
".\Release\Simca.obj"
".\Release\SortStringArray.obj"
".\Release\SpectraLoading.obj"
".\Release\SpectraParameter.obj"
".\Release\StatisticalTOCSY.obj"
".\Release\StdAfx.obj"
".\Release\svm.obj"
".\Release\SVMTest.obj"
".\Release\SVMTrain.obj"
".\Release\SVNCorrection.obj"
".\Release\ULDA.obj"
".\Release\vcaxis.obj"
".\Release\vcaxisgrid.obj"
".\Release\vcaxisscale.obj"
".\Release\vcaxistitle.obj"
".\Release\vcbackdrop.obj"
".\Release\vcbrush.obj"
".\Release\vccategoryscale.obj"
".\Release\vccolor.obj"
".\Release\vccoor.obj"
".\Release\vcdatagrid.obj"
".\Release\vcdatapoint.obj"
".\Release\vcdatapointlabel.obj"
".\Release\vcdatapoints.obj"
".\Release\vcfill.obj"
".\Release\vcfont.obj"
".\Release\vcfootnote.obj"
".\Release\vcframe.obj"
".\Release\vcintersection.obj"
".\Release\vclabel.obj"
".\Release\vclabels.obj"
".\Release\vclcoor.obj"
".\Release\vclegend.obj"
".\Release\vclight.obj"
".\Release\vclightsource.obj"
".\Release\vclightsources.obj"
".\Release\vclocation.obj"
".\Release\vcmarker.obj"
".\Release\vcpen.obj"
".\Release\vcplot.obj"
".\Release\vcplotbase.obj"
".\Release\vcrect.obj"
".\Release\vcseries.obj"
".\Release\vcseriescollection.obj"
".\Release\vcseriesmarker.obj"
".\Release\vcseriesposition.obj"
".\Release\vcshadow.obj"
".\Release\vcstatline.obj"
".\Release\vctextlayout.obj"
".\Release\vctick.obj"
".\Release\vctitle.obj"
".\Release\vcvaluescale.obj"
".\Release\vcview3d.obj"
".\Release\vcwall.obj"
".\Release\vcweighting.obj"
".\Release\Wizard.obj"
".\Release\Worksheet.obj"
".\Release\XGraph2DView.obj"
".\Release\XGraphTestView.obj"
".\Release\Automics.res"
]
Creating command line "link.exe @C:\DOCUME~1\angell\LOCALS~1\Temp\RSP5B10.tmp"

### Output Window

Compiling resources...
Compiling...
StdAfx.cpp
Automatically linking with GuiTk115.dll
Compiling...
AddResource.cpp
AddSpectra.cpp
AixProperty.cpp
Automatically linking with XGraph10.dll
AutoAlignment.cpp
AutoBaseline.cpp
Automics.cpp
AutomicsDoc.cpp
Automatically linking with XGraph10.dll
AutomicsView.cpp
AutoPhase.cpp
BaselineCorrection.cpp
BCMenu.cpp
Automatically linking with XGraph10.dll
BrowerResource.cpp
BucketDlg.cpp
ChartProperty.cpp
ChildFrm.cpp
ColumnStatistics.cpp
CommonUtils.cpp
DirTreeCtrl.cpp
Automatically linking with XGraph10.dll
DisplayVariables.cpp
Automatically linking with XGraph10.dll
Expression.cpp
Generating Code...
Compiling...
ExpressionCal.cpp
FisherCriterion.cpp
font.cpp
font1.cpp
FormatConvert.cpp
FormatDefinition.cpp
FourierTransform.cpp
GuiFolderVisio.cpp
HtmlResource.cpp
ImportFile.cpp
KMean.cpp
KNNClass.cpp
LabelProperty.cpp
LDA.cpp
MainFrm.cpp
MatrixScale.cpp
Automatically linking with XGraph10.dll
ModelSummary.cpp
MoveSpectra.cpp
MSCCorrection.cpp
Automatically linking with XGraph10.dll
mschart.cpp
Generating Code...
Compiling...
msflexgrid.cpp
MyEdit.cpp
MyListCtrl.cpp
MySpin.cpp
NewModel.cpp
OPLS.cpp
OSCCorrection.cpp
PCA.cpp
PeaklistDlg.cpp
PhaseCorrection.cpp
Automatically linking with XGraph10.dll
picture.cpp
Automatically linking with XGraph10.dll
PlotProperty.cpp
PlotSettingsProperty.cpp
PrincipleComp.cpp
ProgDlg.cpp
ProjectStatus.cpp
rowcursor.cpp
RowStatistics.cpp
ScoreProperty.cpp
Simca.cpp
Generating Code...
Compiling...
SortStringArray.cpp
SpectraLoading.cpp
SpectraParameter.cpp
Automatically linking with XGraph10.dll
StatisticalTOCSY.cpp
svm.cpp
SVMTest.cpp
SVMTrain.cpp
SVNCorrection.cpp
ULDA.cpp
vcaxis.cpp
vcaxisgrid.cpp
vcaxisscale.cpp
vcaxistitle.cpp
vcbackdrop.cpp
vcbrush.cpp
vccategoryscale.cpp
vccolor.cpp
vccoor.cpp
vcdatagrid.cpp
vcdatapoint.cpp
Generating Code...
Compiling...
vcdatapointlabel.cpp
vcdatapoints.cpp
vcfill.cpp
vcfont.cpp
vcfootnote.cpp
vcframe.cpp
vcintersection.cpp
vclabel.cpp
vclabels.cpp
vclcoor.cpp
vclegend.cpp
vclight.cpp
vclightsource.cpp
vclightsources.cpp
vclocation.cpp
vcmarker.cpp
vcpen.cpp
vcplot.cpp
vcplotbase.cpp
vcrect.cpp
Generating Code...
Compiling...
vcseries.cpp
vcseriescollection.cpp
vcseriesmarker.cpp
vcseriesposition.cpp
vcshadow.cpp
vcstatline.cpp
vctextlayout.cpp
vctick.cpp
vctitle.cpp
vcvaluescale.cpp
vcview3d.cpp
vcwall.cpp
vcweighting.cpp
Wizard.cpp
Worksheet.cpp
XGraph2DView.cpp
XGraphTestView.cpp
Automatically linking with XGraph10.dll
Generating Code...
Automatically linking with XGraph10.dll
Linking...
   Creating library .\bin/Automics.lib and object .\bin/Automics.exp

### Results

Automics.exe - 0 error(s), 0 warning(s)
```
